# Subcutaneous Splenosis of the Abdominal Wall: Report of a Case and Review of the Literature

**DOI:** 10.1155/2013/454321

**Published:** 2013-01-15

**Authors:** Evangelia Papakonstantinou, Vasileios Kalles, Ioannis Papapanagiotou, Theodoros Piperos, Dimitrios Karakaxas, Vasileios Bonatsos, Konstantinos Tsoumakas, Filotheos Orfanos, Theodoros Mariolis-Sapsakos

**Affiliations:** Department of Surgery, Evgenideion Hospital, University of Athens, P. Perimeni 1-2, Nea Smyrni, 17121 Athens, Greece

## Abstract

Splenosis is a common benign condition that occurs after splenic rupture via trauma or surgery. The mechanism behind splenic cell autotransplantation begins with the splenic rupture, either from trauma or surgical removal. Splenosis is usually found incidentally and, unless symptomatic, surgical therapy is not indicated. Subcutaneous splenosis is an extremely rare form of splenosis, mostly observed in abdominal surgical scars. We report a case of subcutaneous splenosis, as well as a comprehensive review of the literature. In our case, a 43-year-old woman who had splenectomy after traumatic splenic rupture at the age of 7 years old presented for plastic reconstruction of her postoperative scar. Upon surgery, two asymptomatic subcutaneous nodules were incidentally discovered. The presence of splenic tissue was confirmed by the histological study. The nodules were not excised, as the patient was not symptomatic.

## 1. Introduction

Splenosis is a term initially used by Buchbinder and Lipkoff in 1939 in order to describe the heterotopic transplantation of splenic tissue within the abdominal cavity [1]. Splenosis usually occurs after traumatic or surgical rupture of the spleen with autotransplantation of splenic tissue into ectopic sites.

The pathogenesis of splenosis is not well understood and therefore it is not possible to predict when splenic implants will develop. The mechanism behind autotransplantation begins with the splenic rupture, either from trauma or surgical removal. It is presumed that spillage of the damaged splenic pulp into the adjacent cavities begins the seeding process [1–3], while a second mechanism is the hematogenous spread of splenic pulp as suggested by case reports of intrahepatic splenosis [4, 5].

The present report describes a rare case of subcutaneous splenosis in a patient with history of splenectomy, along with a comprehensive review of the literature.

## 2. Case Presentation

**Figure 1:**
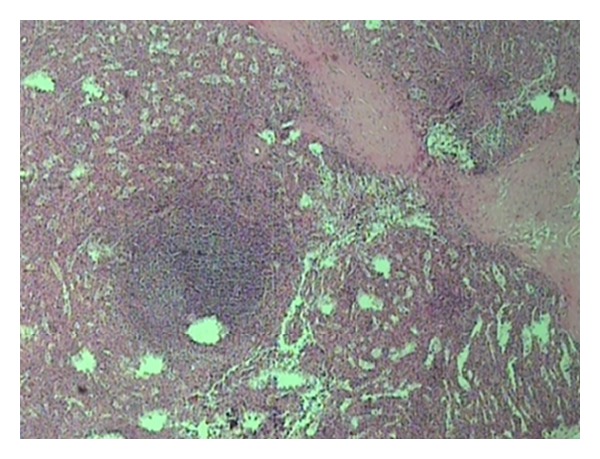
Splenic tissue (H&E). The splenic capsule is thick and trabeculae connective tissue vaguely subdivides the organ.

**Figure 2:**
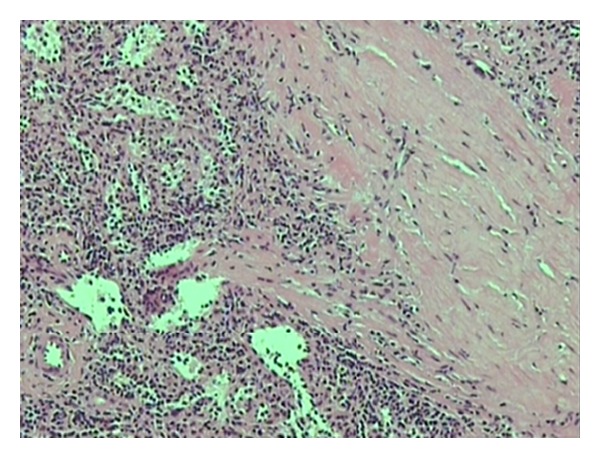
Thick connective tissue containing blood vessels next to red pulp of the spleen.

**Figure 3:**
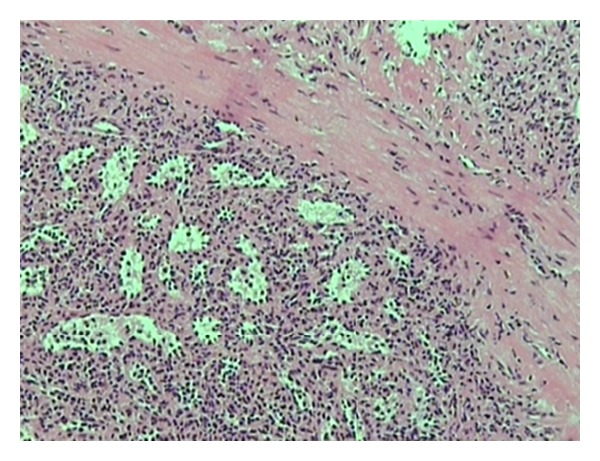
The red pulp consists of a complex network of venous sinuses and the cords of Billroth. The cords contain most of the splenic macrophages. The sinuses have a discontinuous wall which allows traffic of blood cells between cords and sinuses.

**Figure 4:**
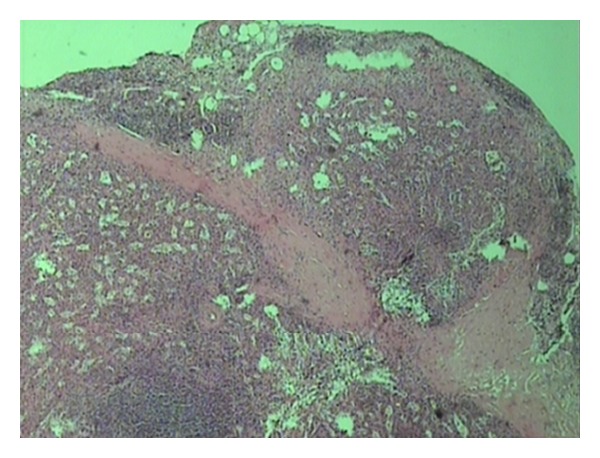
Nodules of splenic tissue clearly separated by the connective tissue of the capsule.

A 43-year-old woman, with a history of splenectomy after traumatic rupture at the age of 7, was referred to our surgical department for plastic reconstruction of a postoperational scar at the left abdominal wall. Preoperative laboratory values were within normal limits. At surgery, we accidentally discovered two subcutaneous nodules that appeared to be immobile and firmly embedded in the subcutaneous fat tissue, whereas the underlying abdominal fascia and muscles appeared intact. The two dark red lesions measured 2.7 cm and 1.7 cm, respectively, and had a hard consistency, resembling with splenic tissue. Biopsy specimens were acquired from both lesions and the histological examination confirmed the presence of splenic tissue with white and red pulp, and no evidence of malignancy (Figure 1). The connective tissue also appeared to contain blood vessels adjacent to splenic red pulp (Figure 2). The red pulp consisted of a complex network of venous sinuses and cords of Billroth, which contain most of the splenic macrophages. The sinuses were lined by a particular type of endothelial cells (known as littoral cells) and had a discontinuous wall, which allows traffic of blood cells between cords and sinuses (Figure 3). The above histological assay reveals that the splenic tissue performed normal splenic function, with absence of Howell-Jolly and Heinz bodies, siderocytes or other abnormal red blood cells. The architecture of the splenic tissue was well developed with nodules of splenic tissue separated by the connective tissue of the capsule (Figure 4). The location of the ectopic splenic tissue strongly favored of the diagnosis of splenosis rather than accessory spleen, a significant clinical dilemma.

It is known that the diagnostic method of choice, before surgery and histological confirmation, is nuclear scintigraphy, a heat-damaged red blood cell scan [20]. As we incidentally discovered the nodules, we did not have the opportunity to perform such an examination, so, we only performed a postoperative ultrasound examination, to examine for more nodules in other regions of the abdominal or thoracic cavity. The ultrasound revealed no other mass. The nodules were not further excised, as no further work up is necessary, once splenosis is confirmed, unless the patient is symptomatic.

## 3. Discussion

Splenosis usually occurs after traumatic or surgical rupture of the spleen with autotransplantation of splenic tissue to ectopic sites. The first known reference to the condition of autotransplantation of splenic tissue following rupture of the spleen was made by Garamella and Hay in 1910 [21]. Albrecht, in 1896 [22], and Schilling [23], in 1907, reported cases of splenic foci, dispersed throughout the peritoneal cavity. Buchbinder and Lipkoff later proposed the term splenosis to describe the presence of multiple peritoneal implants with a widespread location unusual for accessory spleens and with a previous history of trauma to the spleen [1].

 The traumatic etiology of splenosis was first demonstrated by Kreuter in 1920, who reported that controlled, clean, total splenectomy in monkeys was not followed by the appearance of any nodules, while splenic pulp seeded in the peritoneal cavity produced hundreds of implants [24]. Certain studies estimate that splenosis may occur in up to 67% of individuals who suffer traumatic rupture of the spleen and undergo splenectomy [25]. Thus, Most cases of splenosis present as intraperitoneal nodules, especially on the serosa surfaces of the small and large bowel, in the parietal peritoneum, the mesentery, and the diaphragm, which are found either incidentally or after symptomatic complications. Extraperitoneal locations are very rare and mainly include the thoracic cavity after thoracoabdominal trauma with simultaneous splenic rupture and diaphragmatic laceration. Other uncommon locations have also reported, for example, the female genital organs and the brain [26]. Subcutaneous splenosis is also an extremely rare condition, mostly observed in abdominal surgical scars [26]. It presumes a splenic rupture and a path for escape of the splenic tissue from the peritoneum, for example through a laparotomy incision. The splenic cells may successfully implant subcutaneously and produce nodules of splenic tissue that present as a subcutaneous tumor [27].

 The functional capacity of splenic autoimplants is a subject of controversy. There is some suggestion that splenic autoimplants are in fact functional. Specific studies in animals indicate the functional ability of splenic transplants. Rats which developed *Bartonella muris* anemia following splenectomy are protected in 50% of instances if subcutaneous splenic transplants are performed seven weeks prior to splenectomy [28]. Such transplants also reduce the severity of *Trypanosoma lewisi* infection in splenectomized rats [28]. There are cases that reported recurrence of hematological diseases in human patients with splenosis following splenectomy, such as congenital hemolytic anemia, idiopathic thrombocytopenic purpura, and Felty syndrome [15]. In addition, the frequency of postsplenectomy infections appears higher after elective splenectomy than after splenectomy following splenic rapture (where splenosis should be more common), implying a protective rate of splenic implants [15]. The degree of function may relate to the total bulk of splenic tissue present, and studies report that roughly 20–30 cm^3^ of splenic tissue is required to ensure satisfactory immune response against bacteremia [25]. Other experiments concluded that in adult humans the ectopic splenic tissue does not normalize the altered antibody responses observed following splenectomy [29]. Autografted patients, however, had significantly better splenic function than did asplenic patients. This suggests that autotransplantation can restore at least partial reticuloendothelial function. Whether this is sufficient to reduce overwhelming postsplenectomy sepsis rates compared with those in asplenic patients is unknown. Thus, it can be suggested that the small volume of functioning splenic tissue resulting from autotransplantation may provide some degree of immunoprotection [30]. 

Subcutaneous splenosis is extremely rare. Our research revealed 16 published cases, including the present, that have been reported in the English medical literature [2, 6–19]. The clinical features of those cases are summarized in Table 1. Except for two female patients, all the others were male. All the patients had a history of splenic rupture. Ten cases were younger than 30 years old at the time of splenic injury, which is consistent with the fact that splenosis occurs commonly in young men. The time interval between splenic injury and discovery splenosis ranged from 2 to 54 years with a median of 21 years. The long interval seems to reflect very slow growth of the splenic tissue. In 10 cases, autoimplants of splenic tissue were found in old abdominal surgical scars, whereas 6 cases presented at the site of an exit gunshot wound scar. The majority of cases presented as an asymptomatic subcutaneous mass with a reddish blue macroscopic appearance. None of the patients experienced local hemorrhage or pain. All laboratory data in all patients were within normal limits. Almost half of the patients had a single nodule, whereas the other half presented with multiple nodules. The size of the nodules ranged from 0,7 to 8 cm, with a subcutaneous splenic mass of 8 × 5 × 7 cm representing the largest nodule reported to date. However, it is evident that the size of the lesions is usually restricted by limited blood supply, most frequently resulting to nodules less than 3 cm in diameter. 

 Microscopically, subcutaneous splenosis presents as nodular lesions rich in vascular channels. The tissue in splenosis usually reveals distorted architecture with no hilum, a poorly formed capsule and tissue of any shape and size. Most reports describe the tissue as lacking trabecular structures, having less elastic tissue than a normal spleen, and poorly formed or deficient white pulp with normal appearing red pulp and thus present a diagnostic challenge, as the microscopic picture may simulate lymphoproliferative disorder, nodular Kaposi sarcoma, or vascular malformation. Extensive sampling and thorough examination for a typical organoid arrangement of splenic structures of white and red pulp is recommended in such cases. The red pulp usually consists of a complex network of venous sinuses lined by littoral cells and the cords of Billroth form together a pattern not found in vascular tumors and malformations. In other instances, a full complement of red pulp with cords of Billroth and slightly congested venous sinuses, and white pulp showing central arteries with periarteriolar lymphoid sheaths, follicles, and marginal zones are readily recognizable resulting in the appearance similar to that of accessory spleen. The latter would be the main differential diagnosis on solely microscopic grounds. The localization in the subcutis excludes the possibility of an accessory spleen, which occurs in the tail of the pancreas or within the gastrosplenic ligament, reflecting the prenatal development of the abdominal viscera. Furthermore, the presence of a fibrous capsule, sinusoidal vascular channels, and focal lymphoid aggregates is reminiscent of an accessory spleen. 

 The patient's history should be taken under serious consideration when traumatic spleen rupture or splenectomy is noted. Apart from the history, the diagnosis of splenosis can be determined with the aid of laboratory findings, such as the presence of Howell-Jolly bodies in peripheral blood, absence of spherocytosis and with imaging techniques. The diagnosis is difficult using conventional imaging studies such as ultrasound (U/S) and computed tomography (CT), with frequent false positive imaging findings which refer to neoplastic lesions. In most of the cases the diagnosis was made at the operating room. A technetium-99 m sulfur colloid (TC-99 m SC) scintigraphy is the gold standard technique for the diagnosis of splenosis, and it is currently used to monitor splenosis progression (20r). However, technetium-99 m labeled heat-damaged erythrocyte studies have also been shown to provide important information on the existence of ectopic splenic tissue with accuracy [20]. Information on the activity of splenic tissue can be extracted by measuring serum tuftsin levels [3]. Thus, splenosis can be suspected by conventional imaging and confirmed noninvasively by scintigraphy regardless of its ultimate point of implantation [11].

Current clinical practice suggests the preservation of asymptomatic splenosis to preserve immunologic functions and host defenses [14]. Management of splenosis depends on the patient's symptoms. In general it is accepted that asymptomatic implants should not be removed because splenic tissue may be functional and thus useful for the patient. Furthermore, unnecessary excisions of the implants may lead to serious bleeding and damage to the surrounding organs. For symptomatic patients, resection of the implants either by laparoscopy or laparotomy is the treatment of choice [14, 15].

 In conclusion, subcutaneous splenosis is a rare condition that can occur after splenic rupture. It represents a diagnostic challenge that may often lead to confusion and cause the patient to be subjected to needless surgical operations. In all patients with a history of a splenic rupture or splenectomy, subcutaneous splenosis should be considered in the differential diagnosis of a subcutaneous nodule, especially when it occurs in a surgical or gunshot wound scar. Immunohistochemistry is useful in order to confirm the diagnosis.

## Consent

Written informed consent was obtained from the patient for publication of this paper and accompanying images. A copy of the written consent is available for review by the Editor-in-Chief of this journal. 

## Figures and Tables

**Table 1:** Reported cases of subcutaneous splenosis.

Case	Reference	Year	Sex	Age	Age at splenic rupture	Cause of splenic trauma	Site of implantation	Number and size of nodules	Presentation
1	Shaw and Shafi [6]	1932	M	20	N/A	Blunt trauma	Abdominal surgical scar	N/A	Found at autopsy
2	Gill [7]	1944	M	54	52	Gunshot wound	Exit gunshot wound scar	One, 3 × 2 cm	Subcutaneous nodule
3	Raper [8]	1951	M	30	N/A	N/A	Abdominal surgical scar	N/A	Found autopsy
4	Cohen [2]	1954	M	30	9	Splenectomy for ITP	Abdominal surgical scar	One, 1,5 cm	Subcutaneous nodule
5	Baack et al. [9]	1990	F	38	8	Blunt trauma (car accident)	Abdominal surgical scar	One, 2 cm	Enlarging discoloration of abdominal scar
6	Grantham and Clore [10]	1990	M	57	20	Shrapnel injury	Abdominal surgical scar	Multiple, N/A	Incidental finding on CT scan
7	Hibbeln et al. [11]	1995	M	71	17	Gunshot wound	Exit gunshot wound scar	Several, 1-2 cm	Subcutaneous nodules
8	Burvin et al. [12]	1996	M	49	29	Gunshot wound	Exit gunshot wound scar	A few, 0,5–0,8 cm	Subcutaneous nodules
9	Zeebregts et al. [13]	1998	M	63	27	Blunt injury	Abdominal surgical scar	One, 1,5 cm	Subcutaneous nodule
10	Velitchkov et al. [14]	2000	M	47	43	Blunt injury	Abdominal surgical scar	One, 3 × 2 cm	Subcutaneous nodule
11	Khosravi et al. [15]	2004	M	35	14	Splenic injury due to operation for adrenalectomy	Abdominal surgical scar	Two, 0,7 cm and 0,3 cm	Subcutaneous nodules
12	Yeh et al. [16]	2006	M	38	29	Gunshot wound	Exit gunshot wound scar	Multiple, 0,3–2,5 cm	Subcutaneous nodules
13	Boudova et al. [17]	2006	M	23	N/A	N/A	Left inguinal surgical scar	One, 8 × 7 × 5 cm	Subcutaneous nodule
14	Chang et al. [18]	2009	M	38	30	Gunshot wound	Exit gunshot wound scar	One, 2 × 1,5 cm	Subcutaneous nodule
15	Javadrashid et al. [19]	2010	M	40	22	Gunshot wound	Exit wound scar, thorax, abdomen	Multiple, 7 × 4 × 5,5 cm (max. size)	Vague abdominal pain, tachycardia, flushing
16	Present case	2012	F	43	7	Blunt trauma (car accident)	Abdominal surgical scar	Two, 2,7 cm and 1.6 cm	Subcutaneous nodules
